# Ceftazidime and cefepime antagonize 5-fluorouracil’s effect in colon cancer cells

**DOI:** 10.1186/s12885-021-09125-4

**Published:** 2022-01-31

**Authors:** Christina Pfab, Anush Abgaryan, Barbara Danzer, Fatme Mourtada, Weaam Ali, André Gessner, Nahed El-Najjar

**Affiliations:** Institute of Clinical Microbiology and Hygiene, University Hospital Regensburg, 93053 Regensburg, Germany

**Keywords:** Drug-drug interaction, Pharmacodynamics, Antibiotics, Anti-cancer agents, Antagonism, Apoptosis, Colon cancer, 5-fluorouracil, Ceftazidime, Cefepime

## Abstract

**Background:**

Drug-drug interaction (DDI), which can occur at the pharmacokinetics and/or the pharmacodynamics (PD) levels, can increase or decrease the therapeutic or adverse response of a drug itself or a combination of drugs. Cancer patients often receive, along their antineoplastic agents, antibiotics such as ß-lactams to treat or prevent infection. Despite the narrow therapeutic indices of antibiotics and antineoplastic agents, data about their potential interaction are insufficient. 5-fluorouracil (5-FU), widely used against colon cancer, is known for its toxicity and large intra- and inter- individual variability. Therefore, knowledge about its interaction with antibiotics is crucial.

**Methods:**

In this study, we evaluated at the PD levels, against HCT-116 colon cancer cells, DDI between 5-FU and several ß-lactams (ampicillin, benzypenicillin, piperacillin, meropenem, flucloxacillin, ceftazidime (CFT), and cefepime (CFP)), widely used in intensive care units. All drugs were tested at clinically achieved concentrations. MTT assay was used to measure the metabolic activity of the cells. Cell cycle profile and apoptosis induction were monitored, in HCT-116 and DLD-1 cells, using propidium iodide staining and Caspase-3/7 activity assay. The uptake of CFT and CFP by the cells was measured using LC-MS/MS method.

**Results:**

Our data indicate that despite their limited uptake by the cells, CFT and CFP (two cephalosporins) antagonized significantly 5-FU-induced S-phase arrest (DLD-1 cells) and apoptosis induction (HCT-116 cells). Remarkably, while CFP did not affect the proliferation of colon cancer cells, CFT inhibited, at clinically relevant concentrations, the proliferation of DLD-1 cells via apoptosis induction, as evidenced by an increase in caspase 3/7 activation. Unexpectedly, 5-FU also antagonized CFT’s induced cell death in DLD-1 cells.

**Conclusion:**

This study shows that CFP and CFT have adverse effects on 5-FU’s action while CFT is a potent anticancer agent that inhibits DLD-1 cells by inducing apoptotic cell death. Further studies are needed to decipher the mechanism(s) responsible for CFT’s effects against colon cancer as well as the observed antagonism between CFT, CFP, and 5-FU with the ultimate aim of translating the findings to the clinical settings.

**Supplementary Information:**

The online version contains supplementary material available at 10.1186/s12885-021-09125-4.

## Background

Recognition of drug-drug interaction (DDI) potential is considered an integral part of drug development and acknowledged as an important consideration in evaluating of a new molecular entity [1–3]. Considering that conventional known DDI is based on the activation or inhibition of metabolizing enzymes (i.e., CYP family) or interactions with specific transporters (i.e., P-gp or OATPs) [2, 4], the classical preclinical studies evaluating DDI rely, therefore, on testing the involvement of already known transporters and/or metabolizing enzymes. No wonder that other unknown variables, pathways, or mechanisms are the reason for the unexpected DDI, which still represent 12.5% of events that may interfere with optimal clinical outcome. DDI, which causes 4% of cancer death, is of major concern in oncology [5]. This is owing to the inherent toxicity/narrow therapeutic index of anticancer agents, their intra- and inter-individual variability, and their simultaneous administration along with different drugs to cancer patients [6–11]. DDI can be manifested at the pharmacokinetic (PK) and/or the pharmacodynamics (PD) level or a combination of mechanisms of the drug itself or a combination of drugs [12]. At the PD level DDI results in synergistic, additives, or antagonistic effects [7]. For instance, an enhancement of 5-Fluorouracil’s (5-FU) pharmacological effects has been reported when combined with leucovorin, a folinic acid [13, 14], while an increase in cisplatin cytotoxicity has been observed in the presence of furosemide, a diuretic agent [15]. The high susceptibility of cancer patients to infection, associated with their increased risk of mortality and morbidity, necessitates the incorporation of antibiotics as prophylaxis i.e., for immunosuppressed patients, or to treat infections [16]. Several patient studies report on the interaction at the PK levels [17–19] and at the PD levels [19] between methotrexate, an anticancer agent used against different tumors, and different antibiotics. Data on patients treated with other anticancer compounds are insufficient. In vitro, it has been found that combination treatment of ceftazidime and mitoxantrone does not affect the cytotoxic effect of the latter one when tested against leukemic cells [20], while moxifloxacin and ciprofloxacin (fluoroquinolone antibiotics) have been found to enhance cisplatin-induced apoptosis in pancreatic cells [21]. The combination of 5-FU with ciprofloxacin enhances as well cell death induced by the former one in HT-29, colon cancer cells [22]. Intriguingly, antineoplastic agents have been also found to antagonize or potentiate the activity of antibiotics. For instance, it has been reported that the activity of ß-lactam antibiotics against several bacterial strains is enhanced in the presence of 5-FU [23, 24]. Recently, it has been shown that a combination of 5-FU and cefepime (ß-lactam antibiotic) increases the risk of induction of neurotoxic side effects by the latter one through exacerbating early-onset convulsive seizures and eliciting delayed-onset convulsive status epilepticus in mice [25]. Despite the current knowledge about interactions between antibiotics and 5-FU, a gold standard therapy for different types of cancer, the most important of which is colon cancer [26], studies evaluating the effect of ß-lactams on the antineoplastic activity of 5-FU are deficient. Nevertheless, the lack of knowledge of DDI between these drugs does not imply an absence of interaction; it simply means that their potential interaction(s) stems from uncommon pathways that are not yet deciphered. Therefore, this study aimed to evaluate at the PD level using human colon cancer cells and at clinically relevant concentrations*,* potential DDI between 5-FU and different antibiotics widely used in intensive care units: two cephalosporins (ceftazidime (CFT) and cefepime (CFP)), four penicillins (ampicillin, benzylpenicillin, piperacillin, and flucloxacillin), and one carbapenem (meropenem). Our data show significant antagonism, observed at clinically used concentrations, between CFT and CFP and 5-FU. It also highlights for the first time the effectiveness of CFT as an anticancer agent against colon cancer.

## Methods

### Cell culture

Experiments were performed using human colon adenocarcinoma cell lines HCT-116 and DLD-1 cells and normal cell lines (FHs74Int, THP-1, and U937). HCT-116 and DLD-1 cells were kindly provided by Dr. Martin Ehrenschwender (Institute for Clinical Microbiology and Hygiene, University Hospital Regensburg, Germany). U937 and THP-1, two monocyte- like cell lines, were kindly provided by Prof. Dr. André Gessner (Institute for Clinical Microbiology and Hygiene, University Hospital Regensburg, Germany). HCT-116, DLD-1, U937, and THP-1 were cultured in RPMI 1640 (1x)–L-Glutamine medium (Gibco™, Thermo Fisher Scientific, UK) supplemented with 200 mM L-glutamine (PAN-Biotech, Germany) and 10% fetal bovine serum (FBS, PAN-Biotech, Germany). FHs74Int, human normal intestinal cells (ATCC, Manassas, Virginia, USA) were cultured using Hybri-care medium (ATCC, Manassas, Virginia, USA) supplemented with 10% FBS, 30 ng/mL recombinant human epidermal growth factor (Gibco™, Thermo Fisher Scientific, UK), and 1.5 mg/ml sodium bicarbonate (Merck, Darmstatd, Germany). All cells were grown at 37 °C in a humidified atmosphere of 5% CO_2_ and 95% air (Heraeus Incubator, Heraeus Instruments GmbH, Germany). For all experiments, cells were treated with each of the antibiotics or anticancer agent when alone or in combination, at concentrations that fit within their expected ranges in patients [27–31]. Cefepime (CFP) was obtained from Rotexmedica (Trittau, Germany), while ceftazidime (CFT), ampicillin (AMP), benzylpenicillin (BenP), flucloxacillin (FLU), meropenem (MER) and piperacillin (PIP) were from Biozol (Eching, Germany). CFP and 5-FU were prepared in water while CFT was dissolved in methanol (VWR ProLabo Chemicals, Germany).

### Metabolic activity assay

MTT assay was used to assess the proliferation rate of HCT-116 and DLD-1 cells. In this assay, the ability of metabolically active cells to convert tetrazolium salt into a blue formazan product is measured. The absorption of the dissolved formazan was quantified at 540 nm by an ELISA reader (Bio-Rad iMark Microplate Reader). Briefly, cells were seeded (2.5 × 10^4^ cells/well) in 96-well plates and treated with the tested antibiotics [CFP (50 μg/ml), CFT (100 μg/ml)], and the anticancer agent [5-FU (8 μM or 16 μM)] when alone and in combination for 48 h and 72 h.

### Cell cycle distribution

Propidium iodide (PI) staining was used to evaluate the effect of the different treatments on the cell cycle. Briefly, DLD-1 and HCT-116 cells were plated at 5 × 10^4^/ml and treated with CFP (50 μg/ml), CFT (100 μg/ml), 5-FU (8 μM or 16 μM) and their combinations. 48 h and 72 h post-treatment, collected cells were fixed with ice-cold ethanol (70%) and stored at − 20 °C for at least 2 h. Fixed cells, which were washed with PBS and incubated at 37 °C for 1 h with 50 μl RNase (1 mg/ml) (Sigma Aldrich, Germany), were stained for 15 min with PI (Molecular Probes, Eugene, Oregon, USA). Cell cycle was monitored using BD FACSDiva™, and the percentage of cells in the different phases of the cell cycle (pre-G1, G0/G1, S, and G2/M) phases was determined using BD FACSDiva™ Software.

### Caspase-3/7 activity assay

DLD-1 cells were treated with CFT (100 μg/ml) with and without 5-FU (16 μM) while HCT-116 cells were treated with CFT (100 μg/ml) and CFP (50 μg/ml) with and without 5-FU (8 μM). Caspase-3/7 activity was measured 48 h and 72 h post-treatment according to the manufacturer’s protocol (Caspase-Glo-3/7 Assay, Promega Corp, Madison, WI, USA). Briefly, equal volume from treated cells and caspase-3/7 mixture were incubated at room temperature for 3 h, and the luminescence was measured by a microplate reader.

### Determination of antibiotics’ uptake by the cells

The extracellular and intracellular levels of CFP and CFT were determined using LC-MS/MS method [32]. Briefly, DLD-1 and HCT-116 cells, plated in 12 well plates, were treated with CFP (50 μg/ml), CFT (100 μg/ml) alone and in the presence of 5-FU (8 μM or 16 μM) for 2, 4, 6, and 8 h. The extracellular levels of CFP and CFT were measured in the supernatant. To measure the intracellular levels of CFP and CFT, collected cells were lysed by re-suspending each cell pellet in 1 ml of double-distilled water followed by three cycles of freeze/thaw in an icebox at − 80 °C for 10 min proceeded with 2 min at 37 °C. This step was repeated three times, and cell homogenate were vortexed at high-speed prior extraction. 40 μl from each supernatant and cell suspension were combined with 200 μl of internal standard working solution prepared in ice-cold methanol. Samples were then centrifuged, and 100 μl of the supernatant were diluted with 400 μl water in glass vials prior analysis with LC−/MS/MS.

### Statistical analysis

Results from at least three independent experiments were summarized and are expressed as means ± standard error of the mean (SEM). Statistical significance between different treatments was evaluated using one-tailed Student’s *t*-test. The significance level was set to *p* < 0.05 for all experiments.

## Results

### Ceftazidime and cefepime antagonize 5-FU’s effects on the metabolic activity of DLD-1 and HCT-116 cells

**Fig. 1:**
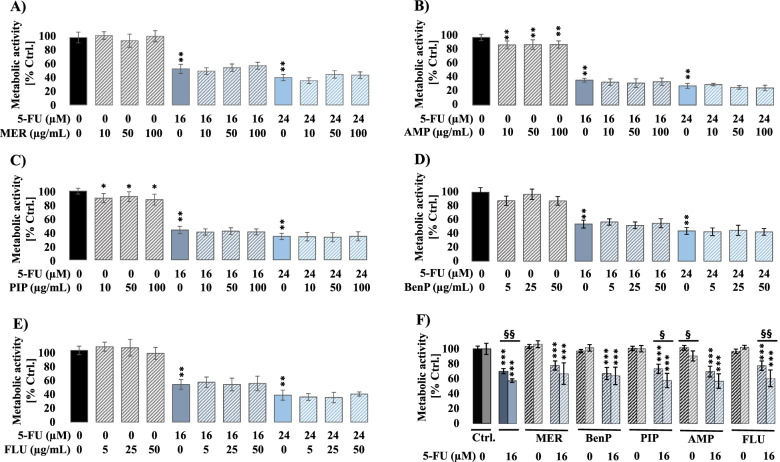
Combination treatment of 5-fluorouracil (5-FU) and the different antibiotics (ampicillin (AMP), benzylpenicillin (BenP), flucloxacillin (FLU), meropenem (MER), and piperacillin (PIP)) does not influence the inhibiting effect induced by 5-FU on HCT-116 cells (**A**-**E**) or on FHs74Int (**F**). The metabolic activity, of HCT-116 cells and FHs74Int, was measured using MTT assay 48 h post-treatment with 5-FU {HCT-116 cells (16 μM and 24 μM); FHs74Int (16 μM)} in the presence and absence of the different antibiotics: MER, AMP and PIP (10, 50 and 100 μg/mL) and BenP and FLU (5, 25 and 50 μg/mL). Results are expressed with respect to their respective control. Each value is the mean ± SEM of three independent experiments. Statistical analysis was performed using one-tailed student *t*-test. *P < 0.05 and **P < 0.01 significant to control untreated cells

**Fig. 2:**
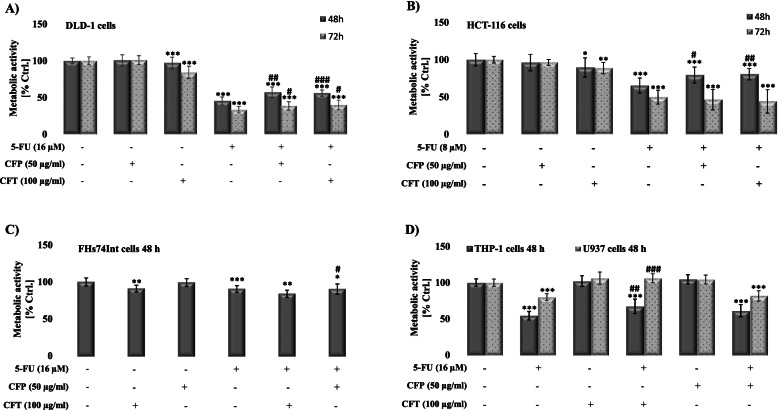
Ceftazidime (CFT) and cefepime (CFP) antagonize 5-fluorouracil (5-FU)’s effect on two colon cancer cell lines (DLD-1 cells (**A**) and HCT-116 (**B**)) but not on human normal intestinal cells (FHs74Int). On monocyte-like cells (THP-1 and U937), on the other hand, only CFT antagonizes 5-FU’s effect. Effect of 5-FU {(8 μM, HCT-116 cells) or (16 μM, DLD-1/FHs74Int/THP-1/U937 cells)}, CFP (50 μg/mL), CFT (100 μg/mL) and their combinations (5-FU/CFT and 5-FU/CFP) 48 h and 72 h post-treatment on the metabolic activity of the different cell lines was measured using MTT assay. Results are expressed with respect to their respective control. Each value is the mean ± SEM of three independent experiments. Statistical analysis was performed using one-tailed student *t*-test. *P < 0.05, *P* < **0.01, and ***P < 0.001 significant to control untreated cells; ^#^*P* < 0.05, ^##^*P* < 0.01, ^###^*P* < 0.001 significant with respect to 5-FU

The effect of combination treatment of 5-FU and several antibiotics (AMP, BenP, FLU, MER, PIP, CFT, and CFP), widely used in intensive care units (ICU), on the metabolic activity of HCT-116 cells was studied using MTT assay. HCT-116 cells were treated for 48 h and 72 h with clinically relevant concentrations of 5-FU [16 μM (therapeutic concentration), and 24 μM (toxic concentration) [28]] and the tested antibiotics [highest, middle, and at the lower expected concentration] in patients [32]. Our data show that combination treatment of AMP, FLU, PIP, MER, and BenP with 5-FU does not affect the inhibition caused by 5-FU on HCT-116 cells (Fig. 1A-E) or on human normal intestinal cells (FHs74Int) (Fig. 1F). CFT and CFP, on the other hand, antagonized 5-FU’s effect. The MTT assay was repeated to confirm and generalize the observed antagonism, using two-cell lines (DLD-1 and HCT-116 cells) showing a differential response to 5-FU. According to Bracht et al. [33], DLD-1 cells are more resistant to 5-FU treatment while HCT-116 cells are more sensitive. Therefore, we selected a concentration that results in a similar killing effect at later time points for each cell line. Consequently, all further analyses were performed using 5-FU at 8 μM and 16 μM for HCT-116 and DLD-1 cells, respectively. Interestingly, while 48 h post-treatment, CFT, and CFP similarly antagonized 5-FU’s effect on HCT-116 and DLD-1 cells (Fig. 2), unpredictably, a significant decrease in the metabolic activity of DLD-1 cells was observed in response to CFT alone (100 μg/ml) 72 h post-treatment. Interestingly, a similar antagonizing effect was also observed on Caco-2 cells, a cell line considered sensitive to 5-FU according to Bracht et al. [33] (Supplemental data Fig. 1).

To evaluate whether a similar effect is observed in normal cells, the effect of CFT and CFP on 5-FU, when alone or in combination, was assessed against FHs74Int (human normal intestinal cells) as well as against U937 and THP-1, two monocyte-like cell lines that differ in their origin and stage of maturation (Fig. 2C-D). Interestingly, 5-FU has a differential inhibitory effect on the metabolic activity of FHs74Int, U937, and THP-1, with THP-1 being the most sensitive. Similar to cancer cells, CFP alone did not affect all three tested normal cells and did antagonize 5-FU’s effect. This data confirms the specificity of CFP’s antagonizing effect on 5-FU to colon cancer cells. CFT, on the other hand, inhibited the metabolic activity of FHs74Int by 8% but did not affect the activity of U937 and THP-1 cells, confirming the safety profile of CFT against normal and immune cells. Interestingly, CFT did not antagonize 5-FU’s inhibitory effect in human normal intestinal cells but reversed partially and completely its inhibitory effect, respectively, in THP-1 cells and U-937 cells. Remarkably, CFT plays a pleiotropic effect on 5-FU, whereas it antagonizes 5-FU’s effect in colon cancer cells, is indifferent to human normal intestinal cells, and exerts a protective role in immune cells. This exciting data merits further investigation.

### Ceftazidime and cefepime antagonize 5-FU’s effects on the cell cycle profile of HCT-116 and DLD-1 cells

To decipher the mechanism(s) of interaction, further mechanistic studies were performed using propidium iodide staining to evaluate the effect of the different treatments on the cell cycle profile of the cells. In HCT-116 cells, a time-dependent increase in cell death was a hallmark of 5-FU’s effect evidenced by the increase in the percentage of cells in the pre-G1 phase {48 h, 3 fold vs. control, *p* < 0.0001; 72 h, 8.2 fold vs. control, *p* < 0.0001). Up to 72 h post-treatment CFT (100 μg/ml) and CFP (50 μg/ml) alone increased slightly the cells in pre-G1 (9.8% ± 0.9, vs. control, *p* < 0.0001) in HCT-116 cells. Interestingly, 48 h but not 72 h post-treatment CFT (100 μg/ml) and CFP (50 μg/ml) antagonized 5-FU-induced increase in pre-G1 phase (Table 1). This data correlated well with the antagonism shown by the proliferation assay in HCT-116 cells by both cephalosporin’s at 48 h but not at 72 h. On the other hand, in DLD-1 cells, 5-FU induced a time-dependent S-phase arrest (*p* < 0.01; vs. control 48 h and 72 h post-treatment). Our findings on 5-FU-induced cell death in both tested cell lines are in accordance with the literature showing that DLD-1 cells are more resistant than HCT-116 cells to 5-FU [33]. CFP and CFT also altered 5-FU-induced changes in the distribution of DLD-1 cells in the different phases of the cell cycle (Table 1). Similar to the proliferation assay, CFT alone affected DLD-1 cells significantly, whereas it induced a time-dependent accumulation of dead cells in the pre-G1 phase (48 h, 1.9 fold vs. control, *p* < 0.01; 72 h, 9.4 fold vs. control, *p* < 0.001). Remarkably, it seems that not only CFT interferes with 5-FU’s mechanism of action, but also the presence of 5-FU antagonizes CFT-induced cell death in DLD-1 cells, as seen by the decrease in CFT-induced accumulation of cells in pre-G1 phase 72 h post-treatment (Table 1). Collectively, the combination of CFT and 5-FU results in an antagonism that affects both drugs. In summary, CFT alone, which significantly inhibited DLD-1 cells, did not significantly affect HCT-116 cells. CFP, on the other hand, had no significant effect on both cell lines but antagonized 5-FU’s effect, although to a lesser extent in comparison to CFT.

**Table 1:** CFT and CFP antagonize 5-FU-induced increase in the percentage of cells in the pre-G1 phase. Propidium iodide staining was used to monitor the cell cycle of HCT-116 cells (A) and DLD-1 cells (B) 48 h and 72 h post-treatment with CFT (100 μg/mL), CFP (50 μg/mL), 5-FU (8 μM for HCT-116 and 16 μM for DLD-1) and their combinations. The distribution of the cells in pre-G1, G0/G1, S, and G2/M phases was determined using FACScan flow cytometry. Their percentages were determined and shown as the mean ± SE of three independent experiments done in duplicate. Statistical analysis was performed using one-tailed student *t*-test. ^a^*P* < 0.05 significant to control untreated cells. ^b^*P* < 0.05 significant with respect to 5-FU. ^c^*P* < 0.05 significant with respect to CFT or CFP

**A) HCT-116**		**0**		**5-FU (8 μM)**	
		***48 h***	***72 h***	***48 h***	***72 h***
**0**	***Pre G1***	4.2 ± 0.7	4.0 ± 0.4	**16.9 ± 2.5**^**(a**)^	**36.7 ± 11.7**^**(a)**^
	***G0/G1***	55.9 ± 4.5	62.7 ± 2.0	**48.4 ± 2.3**^**(a)**^	**39.0 ± 9.5**^**(a)**^
	***S***	11.4 ± 1.7	6.6 ± 1.6	10.6 ± 1.0	6.6 ± 0.7
	***G2/M***	23.4 ± 2.5	21.0 ± 1.4	22.3 ± 2.9	**16.4 ± 2.4**^**(a)**^
**CFP** **50 μg/ml**	***Pre G1***	4.9 ± 1.6	**9.3 ± 2.9**^**(a)**^	**12.6 ± 1.6**^**(a,b,c)**^**(↓)**	**23.2 ± 5.3**^**(a,c)**^
	***G0/G1***	51.7 ± 2.8	**53.8 ± 3.6**^**(a)**^	53.9 ± 3.9	**51.4 ± 4.1**^**(a)**^
	***S***	13.6 ± 1.6	7.4 ± 1.4	**9.7 ± 0.9**^**(c)**^	6.4 ± 0.6
	***G2/M***	24.6 ± 0.9	**23.6 ± 2.2**^**(a)**^	22.5 ± 2.9	**17.5 ± 2.3**^**(a,c)**^
**CFT** **100 μg/ml**	***Pre G1***	**6.4 ± 2.3**^**(a)**^	**9.8 ± 0.9**^**(a)**^	**8.9 ± 1.6**^**(a,b)**^**(↓)**	**35.6 ± 6.4**^**(a,c)**^
	***G0/G1***	53.3 ± 1.7	**56.9 ± 1.3**^**(a)**^	**53.6 ± 3.3**^**(b)**^**(↑)**	**41.6 ± 2.6**^**(a,c)**^
	***S***	12.8 ± 2.9	6.6 ± 1.0	10.8 ± 1.0	**8.0 ± 0.7**^**(b)**^**(↑)**
	***G2/M***	24.4 ± 1.2	**24.3 ± 0.5**^**(a)**^	25.7 ± 5.3	**14.3 ± 4.3**^**(a,c)**^
**B) DLD-1**		**0**		**5-FU (16 μM)**	
		***48 h***	***72 h***	***48 h***	***72 h***
**0**	***Pre G1***	3.2 ± 1.6	3.8 ± 1.1	6.3 ± 2.3	4.7 ± 0.3
	***G0/G1***	53.4 ± 2.5	68.0 ± 2.0	49.3 ± 4.4	69.4 ± 5.0
	***S***	16.4 ± 2.6	8.6 ± 0.9	**32.0 ± 6.1**^**(a)**^	**20.1 ± 2.9**^**(a)**^
	***G2/M***	22.4 ± 2.4	19.1 ± 1.9	**10.3 ± 1.7**^**(a)**^	**5.4 ± 2.7**^**(a)**^
**CFP** **50 μg/ml**	***Pre G1***	2.6 ± 0.3	**9.5 ± 4.1**^**(a)**^	**4.0 ± 1.2**^**(c)**^	**13.6 ± 1.3**^**(a,b)**^**(↑)**
	***G0/G1***	48.8 ± 4.0	**59.6 ± 3.6**^**(a)**^	51.4 ± 9.0	64.6 ± 3.7
	***S***	29.2 ± 8.1	**13.1 ± 3.3**^**(a)**^	31.7 ± 5.3	**17.6 ± 1.2**^**(a)**^
	***G2/M***	18.4 ± 4.3	17.0 ± 3.5	12.8 ± 5.0	**4.3 ± 2.7**^**(a,c)**^
**CFT** **100 μg/ml**	***Pre G1***	**9.3 ± 2.5**^**(a)**^	**40.6 ± 5.2**^**(a)**^	7.1 ± 1.5	**21.0 ± 6.0**^**(a,b,c)**^**(↑)**
	***G0/G1***	52.6 ± 3.3	**38.2 ± 4.3**^**(a)**^	**40.4 ± 3.0**^**(a,b,c)**^**(↓)**	**54.6 ± 7.1**^**(a,b,c)**^**(↓)**
	***S***	14.6 ± 3.2	8.4 ± 0.6	**30.3 ± 7.5**^**(a,c)**^	**10.1 ± 1.3**^**(b)**^**(↓)**
	***G2/M***	**15.7 ± 2.8**^**(a)**^	**12.0 ± 1.6**^**(a)**^	**18.2 ± 3.2**^**(b)**^**(↑)**	**13.7 ± 1.0**^**(a,b)**^**(↑)**

### Combination treatment antagonize apoptosis induction by CFT in DLD-1 and 5-FU in HCT-116 cells

**Fig. 3:**
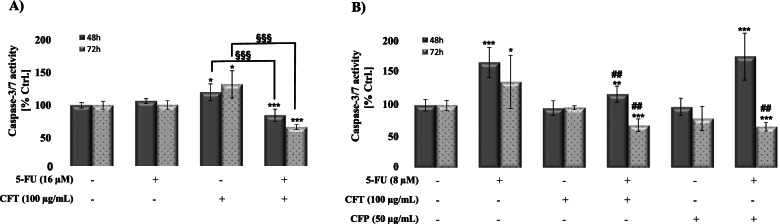
CFT and 5-FU induce Caspase-3/7 assay in DLD-1 cells (**A**) and HCT-116 cells (**B**), respectively. Cells were treated with 5-FU (8 μM for HCT-116 and 16 μM for DLD-1), CFT (100 μg/mL), CFP (50 μg/mL) and their combinations for 48 h and 72 h. Caspase 3/7 activity was measured according to the manufacturer’s protocol. Briefly, a pre-luminescent substrate is cleaved and causes a fluorescent signal proportional to caspase-3/7 activity, which is measured with a microplate reader. Results are expressed with respect to their respective control untreated cells. Standard error of the mean is shown (*n* = 3). Statistical analysis was performed using one-tailed *t*-test. *P < 0.05 and ***P < 0.001: significant with respect to control untreated cells; ^##^*P* < 0.01 and ^###^*P* < 0.001: significant with respect to 5-FU; ^§§^*P* < 0.05 and ^§§§^*P* < 0.001: significant with respect to CFT

To confirm CFT-induced cell death and the antagonism exerted by 5-FU on DLD-1 cells, the activity of caspase-3/7 was measured 48 h and 72 h following treatment with CFT alone, 5-FU alone, and their combination. As seen in Fig. 3, CFT alone increased in a time-dependent manner the activity of caspase 3/7 {20% (48 h, *p* < 0.05), 32.1% (72 h, *p* < 0.05)}, indicating that CFT-induces apoptosis in DLD-1 cells. The increase in caspase 3/7 correlates well with CFT-induced increase in pre-G1 phase of the cell cycle (Table 1). 5-FU alone did not induce caspase 3/7 activity confirming further that in DLD-1 cells and up to 72 h, 5-FU induced S-phase arrest. As seen in the cell cycle analysis, combination of CFT with 5-FU abolished significantly (*p* < 0.001) apoptosis induction by CFT 48 h and 72 h post-treatment (Fig. 3A). Likewise, CFT antagonized 5-FU-induced apoptosis in HCT-116 cells 48 and 72 h post-treatment CFP’s effect was seen only 72 h post-treatment (Fig. 3B).

### CFT and CFP antagonize 5-FU’s effect without entering the cells

Conflicting data exist regarding the ability of cephalosporins to cross the plasma membrane of the cells [34–36]. The fact that significant antagonism was observed between 5-FU and both antibiotics (CFT and CFP), we opted for evaluating whether CFT and CFP are taken up by the cells or whether they induce their effects by residing in the extracellular milieu. The potential uptake of CFT and CFP in the presence and absence of 5-FU was evaluated in DLD-1 and HCT-116 cells up to 8 h post-treatment. HCT-116 and DLD-1 cells were treated with CFT (100 μg/ml) and CFP (50 μg/ml) alone and in combination with either 8 μM or 16 μM 5-FU depending on the cell line. The cells and supernatants were collected up to 8 h post-treatment and processed for analysis by LC-MS/MS [26]. The detected concentration of CFT in the supernatant of DLD-1 and HCT-116 treated cells over 8 h did not decrease significantly (Table 2). Even though up to 8 h of treatment the uptake of CFT by both cell lines is negligible, it is almost 2.5 fold higher in HCT-116 cells (0.047–0.066%) compared to DLD-1 cells (0.019–0.025%). Remarkably, in the presence of 5-FU, the uptake of CFT was significantly decreased after 2 h and 4 h of incubation by 35% (*P* = 0.004) and 50% (*P* = 0.038), respectively in HCT-116 cells while in DLD-1 cells the decrease was only observed at 2 h post-treatment (*P* = 0.032). Remarkably, 8 h post-treatment, a 35% increase in the uptake of CFT (*P* < 0.002) was observed in DLD-1 cells; however, the increased trend measured in HCT-116 cells did not reach significance.

**Table 2:** The uptake of CFT in HCT-116 cells is 2.5 fold higher than in DLD-1 cells. In the presence of 5-FU, CFT’s uptake was significantly decreased in HCT-116 cells, an effect that is less pronounced in DLD-1 cells. Concentrations of CFT when alone or combined with 5-FU, in cell lysates and supernatants (DLD-1 and HCT-116 cells) up to 8 h after treatment were measured by LC-MS/MS [26]. A) CFT (100 μg/ml) ± 5-FU (8 μM) in HCT-116 cells. B) CFT (100 μg/ml) ± 5-FU (16 μM) in DLD-1 cells. *p* < 0.05 (CFT alone vs. in combination), *n* = 3

**A)**	**DLD-1 cells**	**CFT alone (100 μg/ml)**	**CFT (100 μg/ml) + 5-FU (16 μM)**	***P*value** **alone vs. combination**
**2 h**	Supernatant	100.000 ± 2.970	97.736 ± 6.804	0.311
	Lysate	**0.024 ± 0.003**	**0.016 ± 0.0003**	**0.032**
**4 h**	Supernatant	97.073 ± 5.728	98.205 ± 2.551	0.356
	Lysate	0.026 ± 0.002	0.024 ± 0.007	0.428
**6 h**	Supernatant	91.335 ± 7.637	108.665 ± 22.650	0.145
	Lysate	0.033 ± 0.003	0.034 ± 0.004	0.415
**8 h**	Supernatant	98.126 ± 5.940	95.628 ± 5.881	0.297
	Lysate	**0.032 ± 0.001**	**0.049 ± 0.001**	**0.002**
**B)**	**HCT-116 cells**	**CFT alone (100 μg/ml)**	**CFT (100 μg/ml) + 5-FU (8 μM)**	***P*value** **alone vs. combination**
**2 h**	Supernatant	96.900 ± 3.818	93.450 ± 0.636	0.167
	Lysate	**0.047 ± 0.003**	**0.032 ± 0.0002**	**0.004**
**4 h**	Supernatant	105.000 ± 4.168	97.100 ± 12.061	0.172
	Lysate	**0.066 ± 0.012**	**0.033 ± 0.006**	**0.038**
**6 h**	Supernatant	96.750 ± 8.697	95.300 ± 11.915	0.447
	Lysate	0.056 ± 0.001	0.068 ± 0.012	0.139
**8 h**	Supernatant	88.900 ± 8.326	86.850 ± 4.031	0.388
	Lysate	0.053 ± 0.010	0.108 ± 0.027	0.055

Interestingly, in DLD-1 cells, the levels of CFP, when alone or combined with 5-FU did not change in both the supernatant and lysate over 8 h of incubation. On the other hand, in HCT-116 cells, a significant decrease in the levels of CFP in the supernatant was observed (44.1 to 35.9, *p* < 0.05) along with a slight increase in its levels in cell lysates at 6 h and 8 h of treatments. Yet, the increase in cell lysate does not add up for the loss observed in the supernatant, leading to the assumption that in HCT-116 cells, there is non-specific or specific binding of CFP to the plasma membrane (Table 3). This is further supported by the fact that the level of CFP alone or in combination with 5-FU is stable in media alone (Table 4). Interestingly, the uptake of CFP by HCT-116 cells is inhibited significantly in the presence of 5-FU at 6 h and 8 h of incubation. Nonetheless, it seems that in HCT-116 cells, the cell entry of CFP, which is inhibited significantly in the presence of 5-FU at 6 h and 8 h of incubation, is three-fold higher than in DLD-1 cells 6 h and 8 h post-treatment.

**Table 3:** The uptake of CFP in HCT-116 cells is higher than in DLD-1 cells. CFP concentration in combination treatment with 5-FU was not different to single treatment in DLD-1 cells but was inhibited in HCT-116 cells. Concentrations of CFP when alone or combined with 5-FU, in cell lysates and supernatants (DLD-1 and HCT-116 cells) up to 8 h after treatment were measured by LC-MS/MS [26]. A) CFP (50 μg/ml) ± 5-FU (8 μM) in HCT-116 cells. B) CFP (50 μg/ml) ± 5-FU (16 μM) in DLD1 cells. *p* < 0.05 (CFP alone vs. in combination), *n* = 3

**A)**	**DLD-1 cells**	**CFP alone (50 μg/ml)**	**CFP (50 μg/ml) + 5-FU (16 μM)**	***P*value** **alone vs. combination**
**2 h**	Supernatant	50.000 ± 1.697	49.261 ± 7.212	0.440
	Lysate	0.012 ± 0.005	0.027 ± 0.006	0.059
**4 h**	Supernatant	54.187 ± 0.849	51.847 ± 4.879	0.251
	Lysate	0.010 ± 0.0002	0.006 ± 0.006	0.192
**6 h**	Supernatant	45.813 ± 3.818	60.714 ± 9.970	0.069
	Lysate	0.014 ± 0.0001	0.014 ± 0.001	0.138
**8 h**	Supernatant	47.947 ± 12.644	50.082 ± 4.371	0.377
	Lysate	0.012 ± 0.001	0.015 ± 0.013	0.376
**B)**	**HCT-116 cells**	**CFP alone (50 μg/ml)**	**CFP (50 μg/ml) + 5-FU (8 μM)**	***P*value** **alone vs. combination**
**2 h**	Supernatant	44.100 ± 3.395	42.900 ± 5.515	0.409
	Lysate	0.018 ± 0.004	0.016 ± 0.001	0.305
**4 h**	Supernatant	34.500 ± 3.818	27.885 ± 2.440	0.087
	Lysate	0.022 ± 0.005	0.016 ± 0.009	0.242
**6 h**	Supernatant	35.250 ± 1.485	34.400 ± 3.516	0.388
	**Lysate**	**0.050 ± 0.003**	**0.026 ± 0.001**	**0.005**
**8 h**	Supernatant	35.850 ± 1.909	41.000 ± 5.679	0.161
	**Lysate**	**0.032 ± 0.002**	**0.007 ± 0.001**	**0.002**

**Table 4:** Stability of CFT and CFP when alone or combined with 5-FU incubated up to 8 h in media as measured by LC-MS/MS [26]. A) CFP (50 μg/ml) ± 5-FU (8 μM and 16 μM). B) CFT (100 μg/ml) ± 5-FU (8 μM and 16 μM). *P* value (a) (between antibiotic alone and 5-FU (8 μM)); P value (b) (between antibiotic alone and 5-FU (16 μM)), *n* = 3

**A) Stability test**	**CFP (50 μg/mL)**	**CFP (50 μg/mL) + 5-FU (8 μM)**	**CFP (50 μg/mL) + 5-FU (16 μM)**	***P*value (a)**	***P*value (b)**
2 h	49.9 ± 9.5	45.3 ± 6.4	50.9 ± 0.6	0.3	0.5
8 h	48.8 ± 5.3	50.9 ± 7.0	47.2 ± 5.9	0.4	0.4
**B) Stability test**	**CFT (100 μg/mL)**	**CFT (100 μg/mL) + 5-FU(8 μM)**	**CFT (100 μg/mL) + 5-FU(16 μM)**	***P*value (a)**	***P*value (b)**
2 h	104.4 ± 2.5	112.1 ± 4.4	94.6 ± 7.5	0.1	0.1
8 h	103.4 ± 10.8	110.8 ± 11.3	103.4 ± 11.7	0.2	0.3

## Discussion

This study presents data showing the antagonistic effects resulting from the combination of the widely used antineoplastic agent 5-FU and two cephalosporins (CFP and CFT) against colon cancer cell lines. Through different cellular and analytical techniques, it was proven that the antagonism mediated by CFT and CFP is through the activation of intracellular cascades counteracting those induced by 5-FU. The presented data emphasize the necessity of investigating drug-drug interaction (DDI) even for already approved drugs. As albeit the improvement in the prediction of DDI over the past years, unexpected DDI occur. This could be due to several variables that we do not yet understand or accurately measure. For instance, DDI can lead to poor clinical outcome via the involvement of unknown or uncommon metabolic pathways. Unfortunately, it is estimated that 4% of death in cancer patients is due to DDI [5]. Considering the increase in the awareness about toxicities relating to the concomitant presence of anticancer drugs and antibiotics, the number of reported data is still insufficient. Most studies report on the increase in the toxicity, due to interactions at the PK level, of methotrexate (MTX), an anti-folate agent effective against different types of tumors [17, 18, 37, 38], when in combination with different classes of antibiotics such as fluoroquinolones [17], ß-lactams (i.e.: penicillin and cephalosporin) [39, 40], and sulfonamide [34]. Interaction at the PD level has been shown where an enhancement of MTX’s anti-folate effect was observed in the presence of Trimethoprim, an antibiotic agent with anti-folate property [39]. Unfortunately, data on patients treated with other anti-cancer compounds are scarce. In vitro*,* it has been shown that Novobiocin (coumermycin antibiotic) enhances the sensitivity of breast cancer cells to Topotecan by overcoming the breast cancer resistance protein (BCRP)-mediated drug resistance [41]. Furthermore, moxifloxacin and ciprofloxacin (fluoroquinolone antibiotics) enhanced cisplatin-induced apoptosis in pancreatic cells [21]. Ciprofloxacin also enhanced the anti-proliferative effect of 5-FU in HT-29, a colon cancer cell line [22]. On the other hand, the cytotoxic effect of Mitoxantrone against leukemic cells was not affected in the presence of CFT [20]. Altogether, most of the previously published data highlight the increase of cancer cells’ sensitivity to the anticancer treatments in the presence of different classes of antibiotics; yet, this study shows that among the seven tested antibiotics, CFT and CFP (two cephalosporins that belong to the ß-lactams class of antibiotics) antagonized significantly 5-FU-induced cell death in HCT-116 and DLD-1 cells (Figs. 2 and 3). This data indicates that both antibiotics reverse 5-FU’s effect in two different colon cancer cell lines regardless of its mechanism of action (S-phase arrest vs. apoptosis induction) (Table 1). Interestingly, the reported antagonism has not been shown previously.

Many approved drugs exhibit off-target activities that might be considered beneficial. The fact that the safety and formulation of approved drugs are already established, their transition and use against the new targets is facilitated. Another exciting finding from this study is the potent inhibitory effect of CFT against DLD-1 cells, observed at clinically relevant concentration. CFT induced its effect through the induction of apoptosis as evidenced by the increase in caspase-3/7 activity (Fig. 3). This data provide CFT as a potential new drug active against colon cancer cells. Surprisingly, not only CFT antagonized 5-FU’s effect, but also 5-FU antagonized CFT-induced apoptosis in DLD-1 cells (Fig. 3). HCT-116 cells, on the other hand, were less sensitive to CFT, the reason for which remains to be elucidated.

Ample evidence shows the potential of ß-lactam antibiotics as anticancer agents specifically targeting tumor cells [42, 43]. Obviously, not all ß-lactams have inhibitory activities against cancer, as seen in this study that shows an anticancer activity of CFT but not the structurally similar antibiotic CFP [44]. This latter one, however, has been found to inhibit, when complexed with manganese, human breast cancer cells via apoptosis induction [45] and to act, while in combination with radiation, as an efficient radio-sensitizer that increases senescent cell death [46]. Subsequently, the mechanisms by which cephalosporins induce their effect are complex and multifactorial. Despite the potential uptake of cephalosporins by the cells [35], this is still controversial due to their low lipophilicity [36]. 5-FU, on the other hand, is a small molecule that enters the cells and, through the activation of several different cascades, leads to cell death [14, 47, 48]. Consequently, to have a better insight into the mechanism of interaction, we studied the uptake of CFP and CFT by the cells and evaluated whether they are able to cross the plasma membrane of colon cancer cells. LC-MS/MS was used to measure the levels of CFP and CFT inside the cells and in the supernatants up to 8 h of treatment when alone as well as in combination with 5-FU. Our data show that CFP and CFT mainly reside in the extracellular milieu, while their minimal uptake differ between the tested cell lines. It is worth mentioning that the similar average uptake of CFP and CFT by the cells (around 0.02%) in DLD-1 cells is less than in HCT-116 cells (2 folds higher). Nonetheless, it seems that in HCT-116 cells, the cell entry of CFP is significantly inhibited in the presence of 5-FU at 6 h and 8 h of incubation through an unknown mechanism that needs further investigation. Collectively, the data implies that CFP and CFT exert their antagonism through the activation of intracellular cascades counteracting those induced by 5-FU.

## Conclusions

This study reveals data about adverse effects that could result from the combination of the widely used antineoplastic agent 5-FU and two cephalosporins (CFP and CFT). The lack of information regarding potential DDI between 5-FU, CFP, and CFT emphasizes the importance of the current findings. Albeit the new observation, we understand that this in vitro work does not reflect exactly the complexity of what could occur in the human body; therefore, the clinical relevance of the current findings remains to be explored. Nevertheless, further investigations are needed to decipher key players in the observed antagonism. Furthermore, the potent inhibitory effect of CFT through apoptosis induction in DLD-1 cells, a cell line moderately resistant to 5-FU, merits further investigation.

## Supplementary Information

**Additional file 1.**

## Data Availability

The datasets supporting the conclusions of this article are included within the article (and its additional file).

## Abbreviations

5-FU: 5-fluorouracil
AMP: Ampicillin
BCRP: Breast cancer resistant protein
BenP: Benzylpenicillin
CFT: Cefzazidime
CFP: Cefepime
DDI: Drug drug interactions
FLU: Flucloxacillin
ICU: Intensive care units
LC: Liquid chomatography
MER: Meropenem
MTX: Methotrexate
MS: Mass spectrometry
PD: Pharmacodynamics
PI: Propidium iodide
PIP: Piperacillin
PK: Pharmacodynamics
SEM: Standard error of the mean
