# Supplementary material for: Ceftazidime and cefepime antagonize 5-fluorouracil’s effect in colon cancer cells
Source: BMC Cancer. 2022 Jan 31;22:125. doi: 10.1186/s12885-021-09125-4 (PMC8802503; doi:10.1186/s12885-021-09125-4)
Supplement: Supplementary file 1 — Additional file 1. [file 12885_2021_9125_MOESM1_ESM.docx]

**Supplemental data, Results:**

***Ceftazidime (CFT) and cefepime (CFP) antagonize 5-fluoroucacil (5-FU)'s effect on Caco-2 cells***

According to Bracht et al (REF), Caco-2 cells are sensitive to 5-fluorouracil (5-FU); therefore, lower concentrations of 5-FU were selected for this experiment. Caco-2 cells were exposed to 5-FU (4 µM), ceftazidime (CFT, 100 µg/mL), cefepime (CFP, 50 µg/mL), and their combinations for 24 h, the cell viability was investigated using an MTT-assay. The cell viability of Caco-2 cells was reduced significantly (40%) in response to 5-FU, while in combination with either of the antibiotics, a strong antagonism (p ≤ 0.001) was observed. Similar to DLD-1 cells, CFT reduced the viability of Caco-2 cells by 25%.


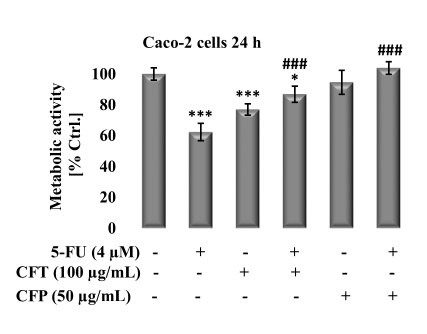


**Figure 1**. Ceftazidime (CFT) and cefepime (CFP) antagonize 5-fluoroucacil (5-FU)'s effect on Caco-2 cells. Effect of 5-FU (4 µM), CFP (50 µg/mL), CFT (100 µg/mL) and their combinations (5-FU/CFT and 5-FU/CFP) 24 h post-treatment on the metabolic activity of Caco-2 cells was measured using MTT assay. Results are expressed with respect to their respective control. Each value is the mean ± SEM of three independent experiments. Statistical analysis was performed using one tailed student *t*-test. ***P<0.001 significant to control untreated cells; ^###^P<0.001 significant with respect to 5-FU.

(REF) Bracht K et al, *Br J Cancer* **103,**340–346 (2010). <https://doi.org/10.1038/sj.bjc.6605780>
